# Some of the Results of Hypertrophic Nasal Catarrh

**Published:** 1891-04

**Authors:** R. O. Cotter

**Affiliations:** President; 572 Mulberry St.


### SOME OF THE RESULTS OF HYPERTROPHIC NASAL CATARRH.

Read at the Meeting of the Macon Medical Society, February 16, 1891.

BY R. O. COTTER, M. D., PRESIDENT.

There has been made, within the past five years, wonderful strides in this hitherto very dark chapter in surgery. As in all new departures, doubtless nasal surgery has been, by some narrow-minded extremists, carried too far. It is also true that men who practice the treatment of twelve or fifteen years ago and who continue to use strong, irritating washes, by means of douches, are rapidly becoming known as "back numbers," among Rhinologists. Just as on the other hand, those who saw away recklessly every little harmless excrescence upon the septum; or apply the Galvano-Cautery to every "Sensitive Area" in cases of hay fever, or to every slight hyperaemia of the mucous membrane of the turbinated bones. This last kind is being "weighed in the balance and found wanting," by the more conservative and successful leaders in this very important branch of surgery.

It seems that for the past two years, especially, the golden mean is being approached. A more correct knowledge of the effects produced by a hypertrophic condition of the nostrils has given us far more satisfactory results in the treatment of disorders of contiguous parts or organs. Such as inflamed eyelids, sore throat, and especially catarrhs of the middle ear.

Why should hypertrophy of the nasal passages cause aural catarrh? Not only by direct extension, but because as the nasal stenosis progresses, the atmospheric pressure back of the hypertrophy becomes lessened, and the whole nostrils become occluded and finally the eustachian tubes participate in the process, and the necessary balance of circulation of air in the tubes to and from the air cells in the middle ear, is deranged. Then we have more or less complete deafness, together with what is most harrassing of all, an intolerable ringing and buzzing in the ears. I believe the most successful aurists now-a-days are those who look for, and generally find, the cause of most cases of middle ear catarrh to be in the nose and naso pharynx. By no means do I now, as I did five years ago, throw away valuable time by trusting alone to simple inflation of the middle ear with the air douche and catheter. If there is nasal hypertrophy, which is a possible cause, I first try to remove it. If an wehandrama or exostosis I use the saw. If a hypertrophy of the turbinated bones, I use the snare or chronite acid. If the ear trouble is not too old, I get far more satisfactory results than I formerly obtained. There is one thing which my own observation has taught me. It is known that the mucous membrane of the middle turbinated is of a more delicate organization than that of the lower turbinated bone, and by far the worst cases of acute tinnitus aurium, which I have noticed, are those which exist with hypertrophy of the middle turbinated bones. Frequently I have found the tinnitus persist after I had sufficiently cauterized the lower turbinated. In some of these cases the hypertrophy of the middle bone was so small that I could not believe at first that it could cause such an amount of trouble, but careful observation has taught me that many of these cases can be quickly relieved by removal of what may seem an insignificant hypertrophy of the middle turbinated bone. These middle hypertrophies being high up and often jammed hard against the septum are much the most difficult to treat. We should be exceedingly careful in cauterizing, or we may cause adhesion between the turbinated and the septum. In some cases I have not hesitated to remove a part of the middle turbinated bone when it was necessary to clear the space for nasal breathing. Nasal stenosis means very great discomfort, and Professor F. H. Bosworth, in his new book, advises the last mentioned course, whenever necessary. In two or three such cases I have had considerable hemorrhage, which continued oozing for two days, greatly to the patient's alarm. I have never yet heard of dangerous hemorrhage from any of these operations upon the turbinated bones.

Adenoid growth in the posterior nares and vault of the pharynx, are common concomitants of hypertrophic catarrh. I am very positive, however, that while we have a very great deal of hypertrophic and atrophic catarrh in this part of the South, the proportion of cases of adenoid vegetations is very much less than I have observed during my visit to the Northern hospitals. These patients are generally troubled with a desire to spit almost constantly. Speaking, or using the voice, is greatly interfered with. Generally the memory is functionally impaired also.

I don't know any disorder, perhaps, whose symptoms yield so beautifully to treatment as do these adenoids when they are removed. I have never found anything so satisfactory as the curette to remove them with Large ones may require the snare. There is another condition which is closely allied to adenoid growths and that is a well-known hypertrophy of the mucous membrane of the naso pharynx. It is especially common in children, but by no means altogether so. It is generally accompanied by buzzing in the ears, and more or less paresis of the muscles of articulation and deglutition. I have often obtained fine results in this condition by the application of a 20 or 40 gr. to the ℥ sol. of arginit, in the manner advised by Dr. A. H. Burk, of New York, some ten years ago.

So much has been written describing the distressing head, brow, and temple aches, the result of direct pressure from hypertrophic catarrh, and they are so well known now, that I will not discuss them here.

Authorities upon nasal diseases assure us that in addition to the troubles which I have referred to, there are certain nasal reflexes associated with it, such as chorea, loss of memory, vertigo, asthma, epilepsy, etc. Of these, I have seen and treated, successfully, vertigo and chorea, and have, in some cases, greatly mitigated and lessened in frequency, attacks of asthma when it was associated with hypertrophic catarrh. I believe that chorea in children is very frequently caused by nasal hypertrophy and hypertrophy of the tonsils. I have seen several such cases and have been successful in relieving them.

572 Mulberry St.

